# A survey of beliefs, attitudes, knowledge, and behaviors about evidence-based practice in physical therapists of Latin America: a cross-sectional study

**DOI:** 10.1038/s41598-024-78703-w

**Published:** 2024-11-09

**Authors:** Marco Antonio Morales-Osorio, Leidy T. Ordoñez-Mora, Héctor Gutiérrez-Espinoza, Felipe Araya-Quintanilla, Ana Bays-Moneo, Robinson Ramírez-Vélez

**Affiliations:** 1 Carrera de Kinesiología, Escuela de Kinesiología, Facultad de Odontología y Ciencias de la Salud, Universidad San Sebastián, Concepción, Chile; 2 Programa de Fisioterapia, Facultad de Salud, Universidad Santiago de Cali, Cali, Colombia; 3 Faculty of Education, Universidad Autónoma de Chile, Santiago, Chile; 4 Escuela de Kinesiología, Facultad de Odontología y Ciencias de la Salud, Universidad San Sebastián, Santiago, Chile; 5 Navarrabiomed, Hospital Universitario de Navarra (HUN), Universidad Pública de Navarra (UPNA), IdiSNA, Pamplona, Spain; 6 CIBER of Frailty and Healthy Aging (CIBERFES), Instituto Carlos III, Madrid, Spain

**Keywords:** Latin American, Evidence-based practice, Evidence-based practice, Attitudes, Knowledge, Barriers, Health care, Medical research

## Abstract

Evidence-based practice (EBP) refers to the integration of scientific evidence into the clinical setting. This study aimed to determine whether Latin American physiotherapists use EBP, analyze the associated factors, barriers, and facilitators, and examine the relationships between these elements in clinical practice. To conduct this cross-sectional study, the web-based questionnaire survey method was chosen. Survey was self-administered questionnaires to assess understanding of various EBP-related terms and determine their knowledge and ability to apply these concepts in clinical practice. The association between attitudes, awareness, and knowledge scores and the demographic data such as age group, years of experience, professional membership registry, and self-reported education was assessed. The questionnaire was distributed to a total of 5000 physical therapists, of which 4099 (82%) responded from seven Latin American countries (Argentina, Bolivia, Chile, Colombia, México, Perú, and Venezuela), between June 1, 2020, and May 31, 2024. Overall, the physical therapists had positive attitudes, beliefs, and interests in EBP. Their educational background, knowledge, and skills related to assessing and interpreting information were associated to the use of EBP. In addition, 53% of respondents indicated that lack of time was the main obstacle to the use of EBP. The top 3 barriers included insufficient time (53%), lack of information resources (20.8%), and lack of research tools (13.6%). Among Latin American physical therapists, individual characteristics workplace, clinical experience, educational background, and demographic characteristics, may behave as facilitators or barriers when performing an EBP. Thus, identifying methods and strategies to support physical therapists in adopting EBP in the Latin American is necessary. The academization of physiotherapy training might change this in the future.

**Supplementary Information:**

The online version contains supplementary material available at 10.1038/s41598-024-78703-w.

## Introduction

Evidence-based practice (EBP) is the implementation of the best research evidence, clinical expertise, and patient preferences in clinical practice. EBP is essential to improve physical therapy (PT) practice, as this strategy includes integration of the best available evidence, clinical expertise, and patient values, preferences, and circumstances related to patient and client management, practice management, and health policy decision making^1^. As a result, EBP has become increasingly vital across various aspects of clinical practice, including physiotherapy^2^.

Numerous studies in the literature have investigated the perception of EBP among PTs^1,3–6^, but only a few such studies are available from Latin American countries. Ramírez-Vélez et al.^7,8^ reported positive attitudes toward EBP, with the majority of PTs believing that the use of research evidence is necessary to their practice. The required skills and knowledge included the ability to phrase and ask a clinical question; search the literature using Boolean operators; and find, read, and critically analyze the research findings using statistical knowledge to select the best and up-to-date evidence at any stage of PT. In similar context, a systematic review found that lack of knowledge, time constraints, and role clarity were the most common barriers reported by PTs^9^. Other barriers included organizational factors such as lack of time and access to research studies as well as negative attitudes toward research and a lack of skills and knowledge kept respondents from implementing EBP^10^.

To date, scarce research has been done regarding attitudes toward and the use of EBP among physical therapists from Latin American countries. Our group detailed Colombian PTs’ attitudes and beliefs regarding EBP in a previous study^11,12^. In the past, the efforts to implement evidence-based practice in PTs have focused on describing potential barriers to the utilization of research evidence among general therapy staff and reducing such barriers^13^. Therefore, if the relationship between the different EBP “actors,” the barriers, and how to avoid them could be understood, we could come closer to accomplishing one important goal (American Physical Therapy Association, 2018): by 2020 PTs would be autonomous practitioners (i.e., independent, self-determined, with professional judgment and action) who, among other things, use EBP.

The primary aim of this study is to determine if Latin American PTs use EBP and more deeply understand the factors, barriers, and facilitators associated with EBP. Our secondary purpose was to describe the associations among the listed elements and characteristics of PTs and their practice environments.

## Materials and methods

### Study design, settings, and participants

To conduct this cross-sectional study, the web-based questionnaire survey method was chosen. This study was conducted in accordance to the Strengthening the Reporting of Observational Studies in Epidemiology (STROBE) guidelines for cross-sectional studies and results have been reported according to the Checklist for Reporting Results of Internet E-Surveys^14^. The target population was practicing physiotherapists from seven Latin American countries (Argentina, Bolivia, Chile, Colombia, México, Perú, and Venezuela), between April 1, 2012, and May 31, 2024. This project was approved by the UMB School of Health Ethics Committee for Research (N° 1008-2012-014) following the ethical standards recognized by the Declaration of Helsinki. Participation in the study was voluntary and no financial compensation was provided for participation in the study.

The World Confederation for Physical Therapy (WCPT) website was used to identify the required sample size (https://world.physio/regions/south-america). The target population was provided from web site WCPT (Chapter South America region) comprises physiotherapy associations from 12 countries/territories. In 2024, the WCPT statistics showed that ~ 11,608 PTs were registered in this chapter from Argentina (*n* = 1208); Brazil (*n* = 3800); Bolivia; Chile (*n* = 1070); Colombia (*n* = 332); Costa Rica (*n* = 3408); Ecuador (*n* = 134); Guatemala (*n* = 180); México (*n* = 621); Perú (*n* = 150); Uruguay (*n* = 410); and Venezuela (*n* = 227). We did not anticipate a high response rate but instead a large enough sample to be indicative within the constraints that non-random samples provide. Considering the geographical and population distribution in the country, it was assumed that about one-third of these PTs (i.e., *n* = 3000) would be working in the 12 countries. Using the Raosoft Sample Size Calculator, the sample size goal for this study was 4020 responses, based on a 95% confidence level, a margin of error of 1%, and an 80% response distribution^15^.

Of the 5000 nonrandomly distributed questionnaires, 82% (4099/5000) were completed and returned. Participants were recruited if they had at least 1 year of clinical experience and were currently working in government hospitals, private clinics, or autonomously. In addition, participants were only recruited if they could read and understand Spanish, which was the questionnaire’s language. The exclusion criteria included a lack of representative data from Costa Rica [*n* = 51, 1,2%]; Ecuador [*n* = 51, 1,2%]; Guatemala [*n* = 15, 0.4%]; and Paraguay [*n* = 41, 1,0%], resulting in 3941 participants included in the analytical sample. Responses were automatically downloaded into SPSS v.21 and remained anonymous.

### Survey instruments

We used a modified version of the Jette et al.^3^ questionnaire, which was developed to survey physiotherapists and was found to be a reliable instrument^3,8,16^, which was validated, and its reliability was confirmed, as reported in a previous paper^17^. An initial invitation and link to the SurveyMonkey^®^ online tool, followed by two fortnightly reminders were distributed via email by the professional bodies, research groups, academies, and snowball convenience sampling utilizing social media and email invitations. The questionnaire was not password protected. The IP addresses of respondents were not recorded or stored, and thus participants’ responses remained anonymous. Before initiating the e-survey (on the front page), the participants were informed of the purpose and context of the study, the data protection rights, and how the results would be used. The front page explained the purpose of the study and assured the participants of the confidentiality of their responses. Consent was obtained through a dichotomous question (yes/no). Only participants who responded affirmatively to this question were included in the study. Multiple entries having the same IP address were not stored and/or limited because the idea was to enable several therapists using the same workplace computer to participate in this study, recognizing that this might lead to multiple entries from the same participant.

The e-survey included 50 closed-ended questions, 18 sociodemographic-related items, and 32 EBP-related items. The original questionnaire contained seven scales that captured information about: (i) attitudes and beliefs about EBP (items 1, 2, 4, and 6–11); (ii) interest in and motivation to engage in EBP (items 3 and 5); (iii) knowledge, and skills related to accessing and interpreting information (items 25–31); (iv) attention to literature, which involved reading research literature, using research literature in making clinical decisions, and the use of medical databases (items 12–14); (v) availability and ability to access information (items 15–17, and 20); (vi) access to and availability of information to promote EBP (items 18, 19, and 21–23); and (vii) perceived barriers to using EBP (item 32). The participants rated their attitudes, beliefs, education, knowledge, and skills related to EBP using a 5-point Likert scale with responses varying from strongly disagree to strongly agree. Items related to access to information required yes/no responses. The following demographic data were collected: gender, age, highest degree earned, current main role in therapy center, hours of work per week, patients seen per day, type of facility, and type of condition and age of most patients (Table 1). The average time to complete the e-survey was 15^2^ min.

**Table 1:** Characteristics of respondents and practice (*n* = 3941).

Characteristics	Argentina	Bolivia	Chile	Colombia	México	Perú	Venezuela	Total sample
Sample, *n* (%)	381 (9.7)	120 (3.0)	378 (9.6)	1456 (36.9)	622 (15.8)	676 (17.2)	308 (7.8)	3941 (100)
Sex, *n* (%)								
Male	220 (57.7)	51 (42.5)	252 (66.7)	308 (21.2)	202 (32.5)	218 (32.2)	112 (36.4)	1363 (34.6)
Female, *n* (%)	161 (42.3)	69 (57.5)	126 (33.3)	1148 (78.8)	420 (67.5)	458 (67.8)	196 (63.6)	2578 (65.4)
Age (y)								
20–29	87 (22.8)	43 (35.8)	128 (33.9)	938 (64.4)	498 (80.1)	174 (25.7)	81 (26.3)	1949 (49.5)
30–39	168 (44.1)	46 (38.3)	163 (43.1)	309 (21.2)	76 (12.2)	254 (37.6)	84 (27.3)	1100 (27.9)
40–49	77 (20.2)	19 (15.8)	56 (14.8)	149 (10.2)	22 (3.5)	157 (23.2)	90 (29.2)	570 (14.5)
50+	49 (12.9)	12 (10.0)	31 (8.2)	60 (4.1)	26 (4.2)	91 (13.5)	53 (17.2)	322 (8.2)
Years licensed, *n* (%)								
< 5	101 (26.5)	45 (37.5)	145 (38.4)	457 (31.4)	504 (81.0)	279 (41.3)	69 (22.4)	1600 (40.6)
5–10	96 (25.2)	32 (26.7)	120 (31.7)	527 (36.2)	48 (7.7)	121 (17.9)	55 (17.9)	999 (25.3)
11–15	64 (16.8)	26 (21.7)	46 (12.2)	202 (13.9)	26 (4.2)	72 (10.7)	58 (18.8)	494 (12.5)
> 15	120 (31.5)	17 (14.2)	67 (17.7)	270 (18.5)	44 (7.1)	204 (30.2)	126 (40.9)	848 (21.5)
Highest degree, *n* (%)								
bachelor’s degree	308 (80.8)	60 (50.0)	117 (31.0)	1094 (75.1)	510 (82.0)	357 (52.8)	182 (59.1)	2628 (66.7)
Specialization	55 (14.4)	30 (25.0)	92 (24.3)	227 (15.6)	30 (4.8)	142 (21.0)	112 (36.4)	688 (17.5)
Master’s	15 (3.9)	28 (23.3)	151 (39.9)	130 (8.9)	80 (12.9)	157 (23.2)	10 (3.2)	571 (14.5)
Doctorate	3 (0.8)	2 (1.7)	18 (4.8)	5 (0.3)	2 (0.3)	20 (3.0)	4 (1.3)	54 (1.4)
Hours of work per week, *n* (%)								
< 20	32 (8.4)	36 (30.0)	38 (10.1)	172 (11.8)	342 (55.0)	104 (15.4)	72 (23.4)	796 (20.2)
20–30	80 (21.0)	46 (38.3)	48 (12.7)	308 (21.2)	118 (19.0)	137 (20.3)	70 (22.7)	807 (20.5)
31–40	133 (34.9)	15 (12.5)	54 (14.3)	423 (29.1)	74 (11.9)	251 (37.1)	90 (29.2)	1040 (26.4)
> 40	136 (35.7)	23 (19.2)	238 (63.0)	553 (38.0)	88 (14.1)	184 (27.2)	76 (24.7)	1298 (32.9)
Patients per day, *n* (%)								
< 5	16 (4.2)	56 (46.7)	140 (37.0)	279 (19.2)	456 (73.3)	162 (24.0)	87 (28.2)	1196 (30.3)
5–10	120 (31.5)	44 (36.7)	95 (25.1)	443 (30.4)	108 (17.4)	257 (38.0)	140 (45.5)	1207 (30.6)
11–15	89 (23.4)	10 (8.3)	74 (19.6)	360 (24.7)	24 (3.9)	169 (25.0)	54 (17.5)	780 (19.8)
> 15	156 (40.9)	10 (8.3)	69 (18.3)	374 (25.7)	34 (5.5)	88 (13.0)	27 (8.8)	758 19.2)
No. of physical therapists at facility, *n* (%)								
< 5	185 (48.6)	106 (88.3)	223 (59.0)	624 (42.9)	500 (80.4)	408 (60.4)	244 (79.2)	2290 (58.1)
5–10	100 (26.2)	12 (10.0)	76 (20.1)	474 (32.6)	82 (13.2)	130 (19.2)	38 (12.3)	912 (23.1)
11–15	26 (6.8)	2 (1.7)	29 (7.7)	113 (7.8)	14 (2.3)	54 (8.0)	17 (5.5)	255 (6.5)
> 15	70 (18.4)	0 (0.0)	50 (13.2)	245 (16.8)	26 (4.2)	84 (12.4)	9 (2.9)	484 (12.3)
Setting, *n* (%)								
Urban	356 (93.4)	103 (85.8)	347 (91.8)	1320 (90.7)	488 (78.5)	590 (87.3)	271 (88.0)	3475 (88.2)
Rural	8 (2.1)	8 (6.7)	13 (3.4)	75 (5.2)	72 (11.6)	42 (6.2)	19 (6.2)	237 (6.0)
Suburban	17 (4.5)	9 (7.5)	18 (4.8)	61 (4.2)	62 (10.0)	44 (6.5)	18 (5.8)	229 (5.8)
Condition for majority of patients treated, *n* (%)								
Orthopedic	195 (51.2)	56 (46.7)	204 (54.0)	383 (26.3)	352 (56.6)	467 (69.1)	205 (66.6)	1862 (47.2)
Neurological	56 (14.7)	26 (21.7)	32 (8.5)	109 (7.5)	54 (8.7)	72 (10.7)	26 (8.4)	375 (9.5)
Cardiovascular/respiratory	60 (15.7)	9 (7.5)	76 (20.1)	715 (49.1)	36 (5.8)	45 (6.7)	22 (7.1)	963 (24.4)
Pediatric/neonatal	2 (0.5)	1 (0.8)	1 (0.3)	65 (4.5)	4 (0.6)	6 (0.9)	0 (0.0)	79 (2.0)
Geriatric	25 (6.6)	12 (10.0)	26 (6.9)	47 (3.2)	58 (9.3)	48 (7.1)	9 (2.9)	225 (5.7)
Sports rehabilitation	34 (8.9)	14 (11.7)	24 (6.3)	45 (3.1)	94 (15.1)	22 (3.3)	42 (13.6)	275 (7.0)
Others	9 (2.4)	2 (1.7)	15 (4.0)	35 (2.4)	24 (3.9)	16 (2.4)	4 (1.3)	105 (2.7)
No patient care	0 (0.0)	0 (0.0)	0 (0.0)	39 (2.7)	0 (0.0)	0 (0.0)	0 (0.0)	39 (1.0)
Patient Care, *n* (% of total work time)								
0–25%	28 (7.3)	9 (7.5)	22 (5.8)	135 (9.3)	39 (6.3)	61 (9.0)	34 (11.0)	328 (8.3)
25–50%	41 (10.8)	16 (13.3)	62 (16.4)	209 (14.4)	94 (15.1)	102 (15.1)	37 (12.0)	561 (14.2)
50–75%	123 (32.3)	46 (38.3)	95 (25.1)	415 (28.5)	183 (29.4)	202 (29.9)	87 (28.2)	1151 (29.2)
+ 75%	189 (49.6)	49 (40.8)	199 (52.6)	697 (47.9)	306 (49.2)	311 (46.0)	150 (48.7)	1901 (48.2)
Researcher, *n* (% of total work time)								
0–25%	314 (82.4)	93 (77.5)	314 (83.1)	1122 (77.1)	507 (81.5)	527 (78.0)	221 (71.8)	3098 (78.6)
25–50%	59 (15.5)	25 (20.8)	47 (12.4)	273 (18.8)	93 (15.0)	119 (17.6)	75 (24.4)	691 (17.5)
50–75%	1 (0.3)	0 (0.0)	10 (2.6)	30 (2.1)	11 (1.8)	12 (1.8)	5 (1.6)	69 (1.8)
+ 75%	7 (1.8)	2 (1.7)	7 (1.9)	31 (2.1)	11 (1.8)	18 (2.7)	7 (2.3)	83 (2.1)
Teacher, *n* (% of total work time)								
0–25%	328 (86.1)	100 (83.3)	313 (82.8)	1214 (83.4)	525 (84.4)	554 (82.0)	269 (87.3)	3303 (83.8)
25–50%	31 (8.1)	12 (10.0)	50 (13.2)	160 (11.0)	67 (10.8)	85 (12.6)	20 (6.5)	425 (10.8)
50–75%	12 (3.1)	7 (5.8)	7 (1.9)	43 (3.0)	17 (2.7)	20 (3.0)	10 (3.2)	116 (2.9)
+ 75%	10 (2.6)	1 (0.8)	8 (2.1)	39 (2.7)	13 (2.1)	17 (2.5)	9 (2.9)	97 (2.5)
Certified specialist (yes), *n* (%)	168 (44.1)	38 (31.7)	120 (31.7)	398 (27.3)	48 (7.7)	164 (24.3)	94 (30.5)	1030 (26.1)
Clinical instructor (yes), *n* (%)	97 (25.5)	43 (35.8)	175 (46.3)	340 (23.4)	130 (20.9)	182 (26.9)	99 (32.1)	1066 (27.0)
Professional membership register (yes), *n* (%)	160 (42.0)	29 (24.2)	78 (20.6)	349 (24.0)	50 (8.0)	87 (12.9)	97 (31.5)	850 (21.6)
Participate in continuing education (yes), *n* (%)	327 (85.8)	114 (95.0)	312 (82.5)	1130 (77.6)	354 (56.9)	587 (86.8)	224 (72.7)	3048 (77.3)

### Data analyses

Response frequencies for the survey questions were determined and displayed in tabular and graphic formats, using Microsoft Excel (Microsoft Corp, Redmond, Washington, DC, USA) and IBM SPSS 26.0 (International Business Machines Corporation, Armonk, NY, USA) software. All data were de-identified to maintain confidentiality. Descriptive statistics were used to analyze frequencies and distributions. Before examining the associations between variables, some categories were collapsed for use as dependent measures in logistic regression analyses. Using a similar strategy to that of Jette et al.^3^ and Ferreira et al.^16^, for items with a four-point Likert scale, the “Strongly Agree” and “Agree” categories were combined, as well as the “Strongly Disagree” and “Disagree” categories, so that responses fell into one of two categories: “Agree” or “Disagree”. For the items with a “Yes/No/Do Not Know” choice set, the “Do Not Know” category was combined with the “No” category, based on the belief that a lack of knowledge regarding EBP topics is similar to not performing EBP. For items categorized by the number of times, the lowest categories (≤ 1 and 2–5) were distinguished from the higher categories (6–10, 11–15, and ≥ 16), and combined into “Poor” for the lower values and “Good” for the high values. For items that were designed to examine the degree of understanding of research terms, the “Understand Completely” and “Understand Somewhat” categories were combined so that a 2-category response was obtained: “Understand Completely” and “Understand at Least Somewhat *plus* Do Not Understand”. Lastly, in the barriers item, the PTs’ choices were collapsed into “Present” (if the PT chooses 1st, 2nd, or 3rd ) or “Absent” (no barrier choice). After item categories were collapsed, logistic regression tests were used to assess the association between the demographics and research-related items (attitudes and beliefs about EBP; interest in and motivation to engage in EBP; educational background, knowledge, and skills related to accessing and interpreting information; the level of attention to and use of the literature; access to and availability of information to promote EBP; and perceived barriers to using EBP). An α of 0.05 was used to define whether a model needed to be reported. Odds ratios (ORs) and their 95% CIs were determined for each level of the independent variables controlling for the country. CIs including 1.0 were considered not statistically significant.

## Results

### Participants’ characteristics

Table 1 summarizes the participants’ characteristics. Most of the PTs that completed the questionnaire were Colombian (36.9%), female (65.4%), within the 20–29-year age group (49.5%), and had obtained a valid PT working license < 5 years ago (40.6%). Furthermore, although the majority only had a baccalaureate degree (61.1%), they showed interest in pursuing a higher academic degree in the future (66.7%). One out of five respondents (21.6%) reported belonging to a practice-oriented organization, possessing specialist certifications (26.1%), and participating, at least once per year, in continuing education courses (72.7%). Regarding their practice, most of the PTs worked more than 40 h per week (32.9%), focusing their time on taking care of the majority of patients treated for orthopedic and cardiovascular/respiratory conditions (47.2% and 24.4%, respectively), and only 1% reported making patient care a low priority. Typically, the PTs treated 11–15 adult patients per day, practicing in settings with fewer than 5 PTs on staff (30.6% and 58.1%, respectively). The majority worked in an urban environment (88.9%) and focused their time on patient care (48.2%), leaving research and teaching as a low priority (78.6% and 83.8% within the 0–25% range, respectively).

### Attitudes and beliefs

**Fig. 1:**
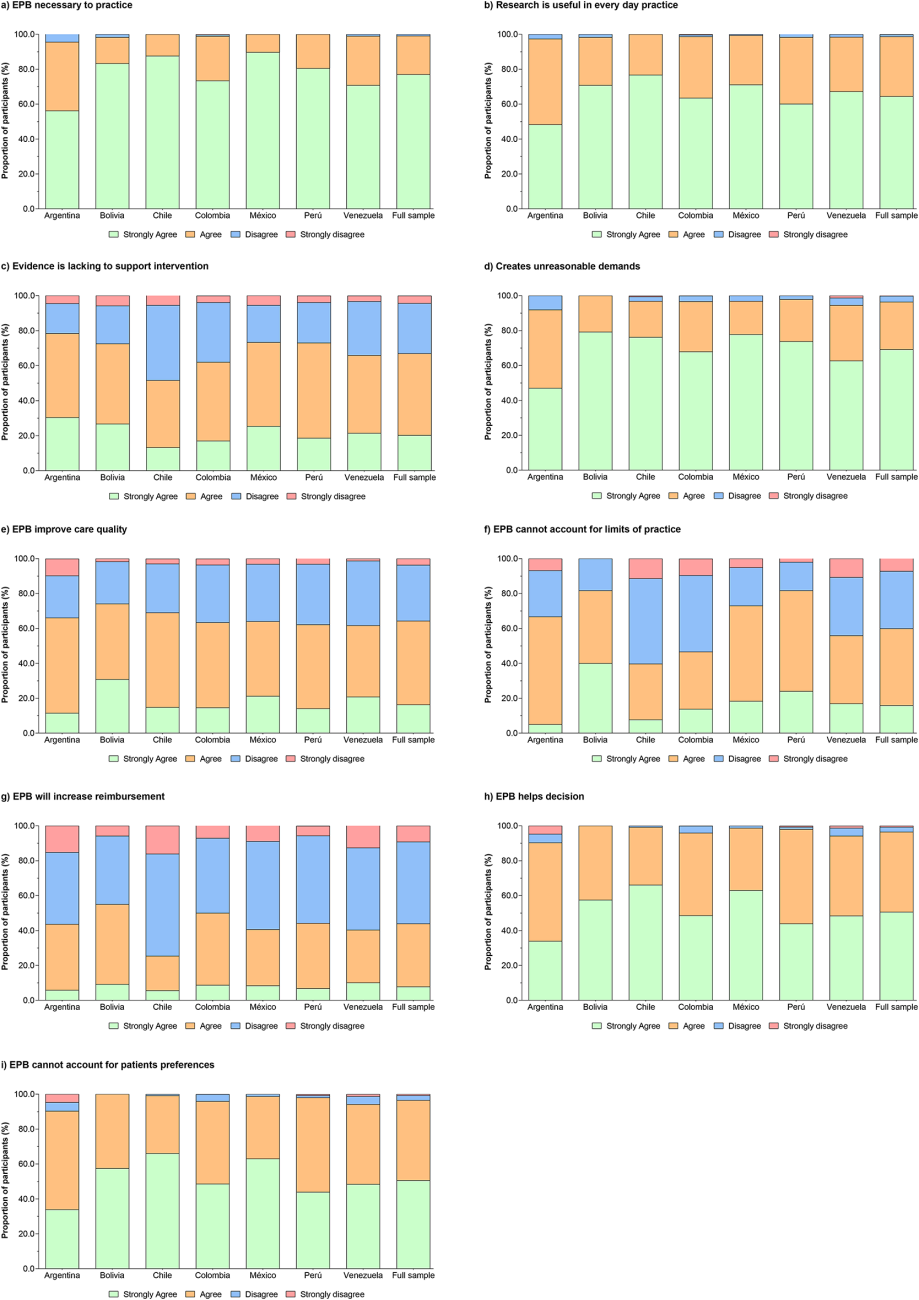
Self-reported attitudes and beliefs about EBP.

The respondents stated that they generally held positive attitudes and beliefs regarding EBP, with a majority contending that they strongly agreed or agreed that EBP is necessary (99%), literature is useful to practice (98%), EBP improves the quality of patient care (64.3%), and evidence helps in decision making (96%). One-fourth percent of the respondents stated they either disagreed or strongly disagreed that using evidence in practice places unreasonable demands on them. The respondents were diverse in their beliefs about whether there was a lack of strong evidence to support aspects of their practice. 40% stated that they disagreed or strongly disagreed, and 60% stated that they agreed or strongly agreed with the statement. Figure 1 shows the distribution of responses related to attitudes and beliefs about EBP.

Regarding the statistically significant associations between PTs’ characteristics and their perceived attitudes and beliefs about EBP, where associations were found, those therapists who had been licensed for fewer years were more likely to say they agreed that “EBP is necessary to practice” (OR = 4.5, 95%CI 1.9–11.0, *P* = 0.002), “reimbursement will increase with the use of EBP” (OR = 2.6, 95%CI 2.2–3.1, *P* < 0.001), and “EBP helps decision making” (OR = 5.4, 95%CI 3.4–8.1, *P* < 0.001). Also, PTs in the 20–29 age group, those who had less than 5 years since licensure, and those with 20–30 h working hours/week were, respectively, 1.7, 2.2, and 2.4 times (95%CI 1.4–2.3, *P* < 0.001; 1.8–2.6, *P* < 0.001; 2.0–2.8, *P* < 0.001) more likely to agree that “evidence is lacking to support interventions,” in comparison with the 30–49 age group, those who had less than 5 years since licensure, and those who worked less than < 20 h or in the range of 31–40 h/week (*P* < 0.001). Table S1 summarizes the sub-group logistic regression information.

### Self-reported education, knowledge, and skills

**Fig. 2:**
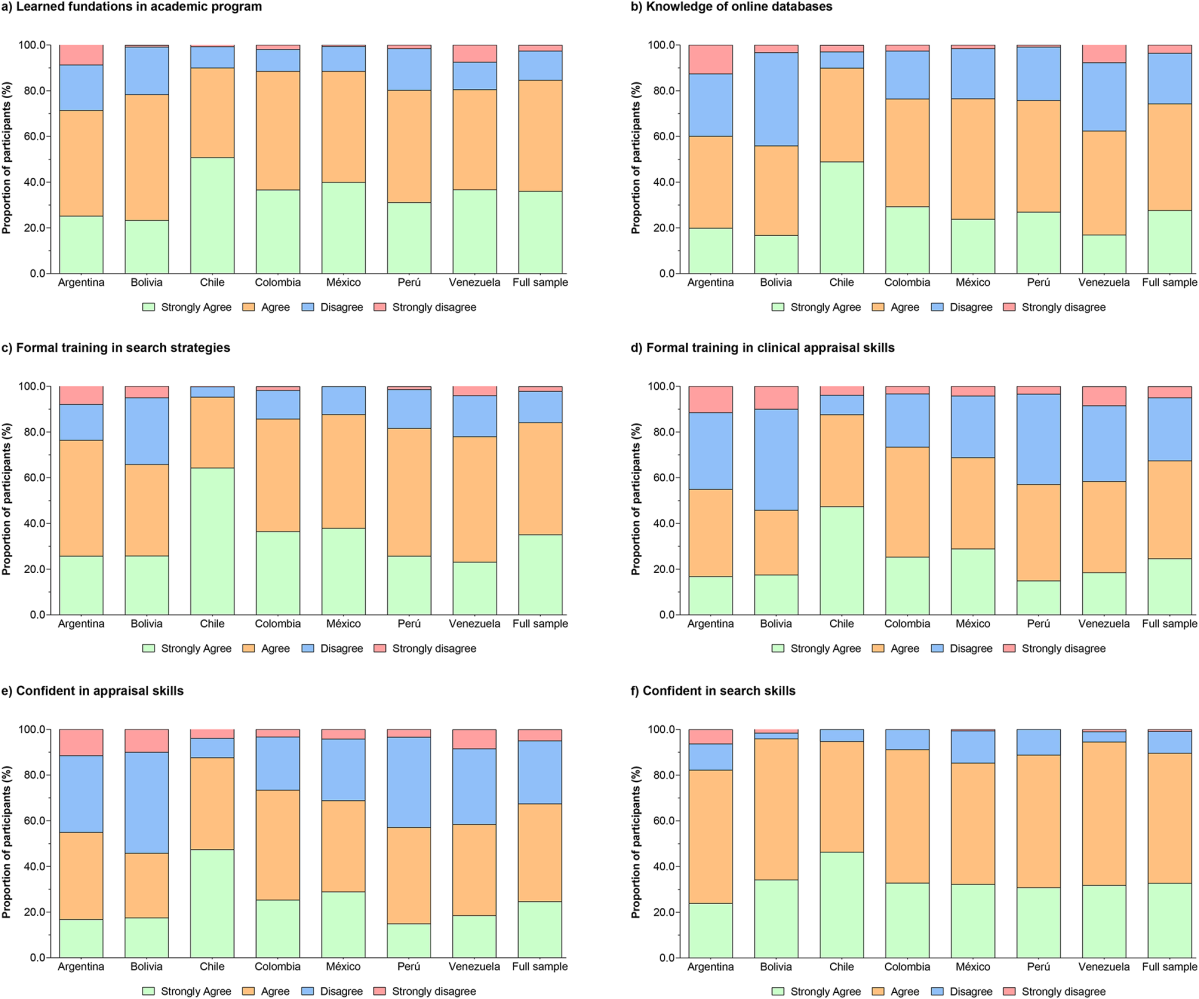
Self-reported education, knowledge, and skills.

The respondents were diverse in expressing whether or not they had completed educational sessions either in school or through continuing education on EBP or search strategies. 48% agreed, and 27% strongly agreed that they had engaged in educational sessions in the foundations of EBP or search strategies, respectively. 67% of the respondents agreed or strongly agreed that they were confident they had search skills, and 84% of the respondents agreed or strongly agreed that they knew how to use databases such as MEDLINE and CINAHL. 24% of respondents stated that they strongly agreed that they were educated in critical appraisal of the research literature, and 56% of the respondents agreed that they were confident in their abilities in this skill. Figure 2 shows the distribution of responses related to education, knowledge, and skills associated with EBP.

Concerning the sub-group items self-reported education, knowledge, and skills, respondents stated that they held generally positive attitudes and beliefs regarding EBP. For example, PTs who had been licensed for 5–10 years and who were younger were more likely to agree that they had “learned foundations in their academic program,” were “familiar with online databases,” had “formal training in search strategies,” and were more confident in search skills. A similar comparison was found in all items for self-reported education, knowledge, and skills, where PTs who “participated in continuing education courses” and were on the “professional membership registry” were (OR) 1.5–4.8 and 1.3–5.4 times, respectively, more likely to agree (*P* < 0.001). Clinical instructors were (OR) 1.9–2.5 times more likely than non-clinical instructors to give a positive response to the six sub-group items for self-reported education, knowledge, and skills (*P* < 0.001). Table S2 summarizes the sub-group logistic regression information.

### Self-reported knowledge of specific terms

**Fig. 3:**
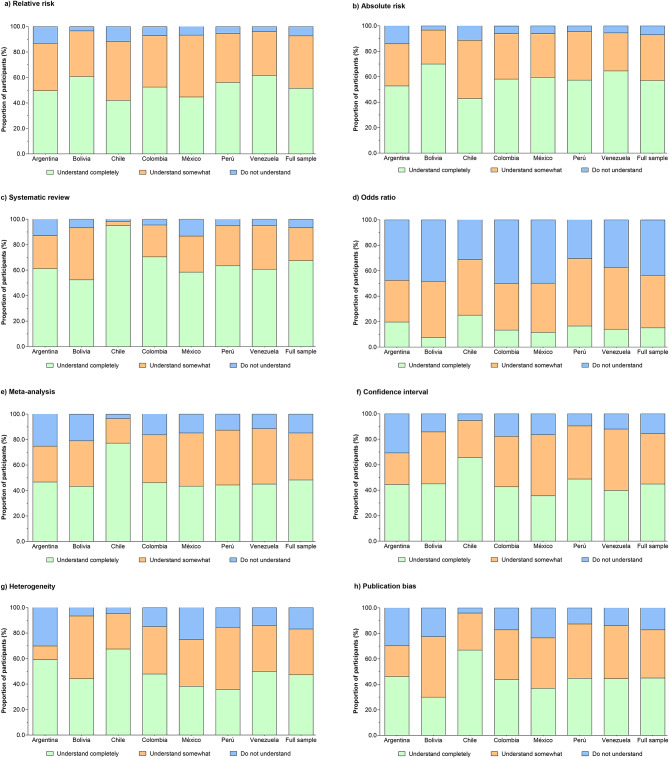
Self-reported knowledge of specific terms.

The results revealed that PTs had diverse knowledge regarding the terms related to EBP. The PTs largely understood the following terms: systematic review (67.5%), absolute risk (57.1%), and relative risk (51.6%). However, many PTs did not understand the following terms: odds ratio (43.5%), publication bias (17.3%), heterogeneity (16.8%), confidence interval (15.6%), and meta-analysis (14.8%), Fig. 3. Respondents who understood the diverse terms related to EBP were more likely to be “male” therapists (range OR = 1.6–3.0), “participate in continuing education courses” (range OR = 1.8–2.7), belong to the “professional membership register” (range OR = 2.2–3.7), and belong to “clinical instructors” (range OR = 1.6–3.4), in comparison with their peers (*P* < 0.001). Table S3 summarizes the sub-group logistic regression information.

### Self-reported attention to the literature

**Fig. 4:**
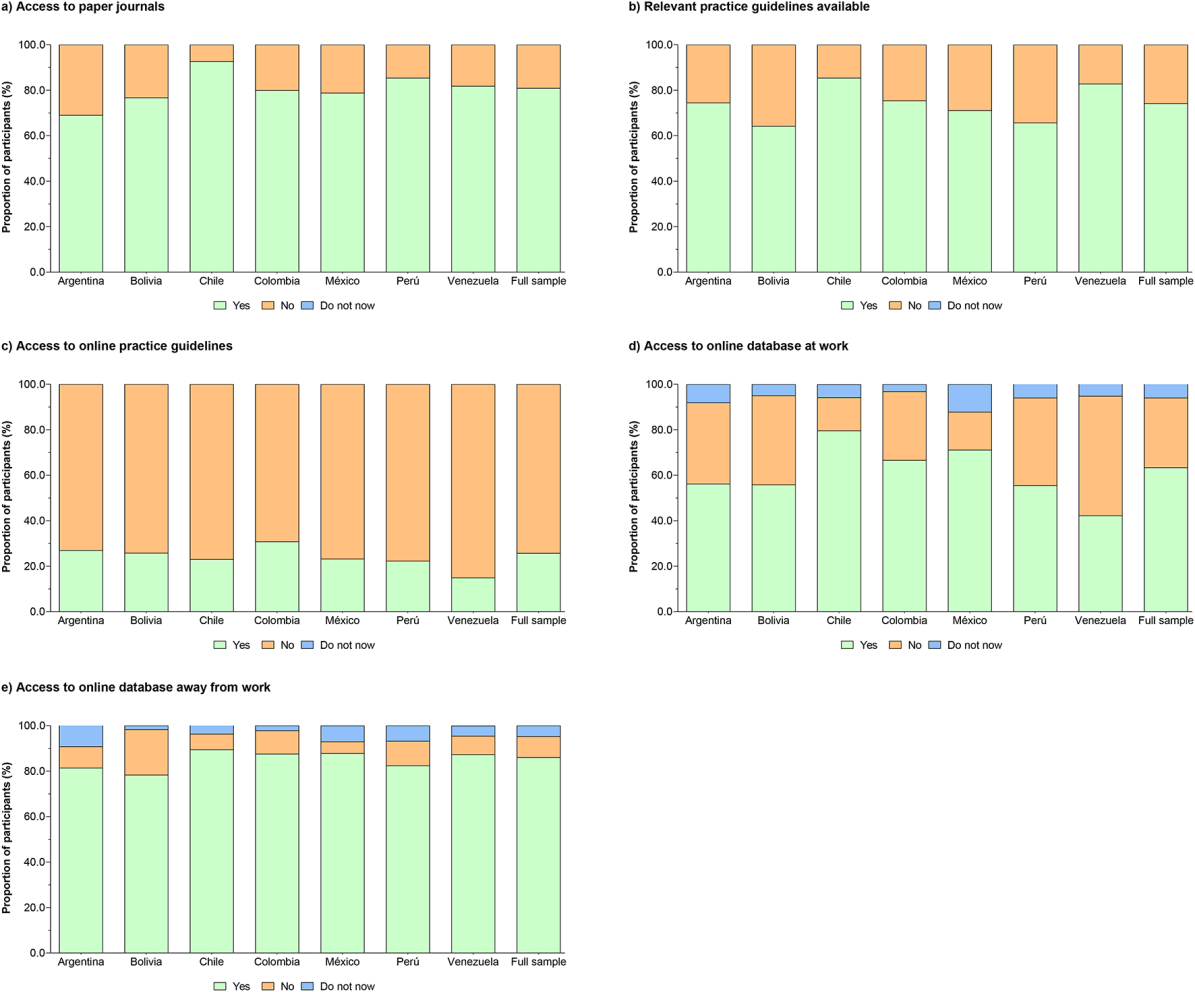
Self-reported attention to literature.

On average, per month, the majority of PTs read 2–5 articles related to their clinical practice (54%); used 2–5 articles in the process of clinical decision-making (47.2%); and performed 2–5 data searches for practice-relevant literature (41%). Figure 4 shows the distribution of responses related to attention to the literature. Once the adjustment was performed by country, the majority of respondents who reported reading 2–5 articles related to their clinical practice were those who had been licensed for “less 5 years” OR 3.7 (95% CI 3.1–4.5), were in the “younger group” OR 3.4 (95% CI 2.7 to 4.4), “worked for a minimum of 31 − 40 hours per week” OR 2.9 (95% CI 2.4 to 3.5), and had a patient load of “5–10 patients per day” OR 3.2 (95% CI 2.6 to 3.9), in comparison with their peers (*P* < 0.001). Similarly, PTs who used the literature in decisions per month were more likely to be in the “male” group OR 1.5 (95% CI 1.3 to 1.8) and belong to the “professional membership register” OR 1.2 (95% CI 1.0 to 1.4; *P* < 0.001). Table S4 summarizes the sub-group logistic regression information.

### Self-reported access to and availability of literature

**Fig. 5:**
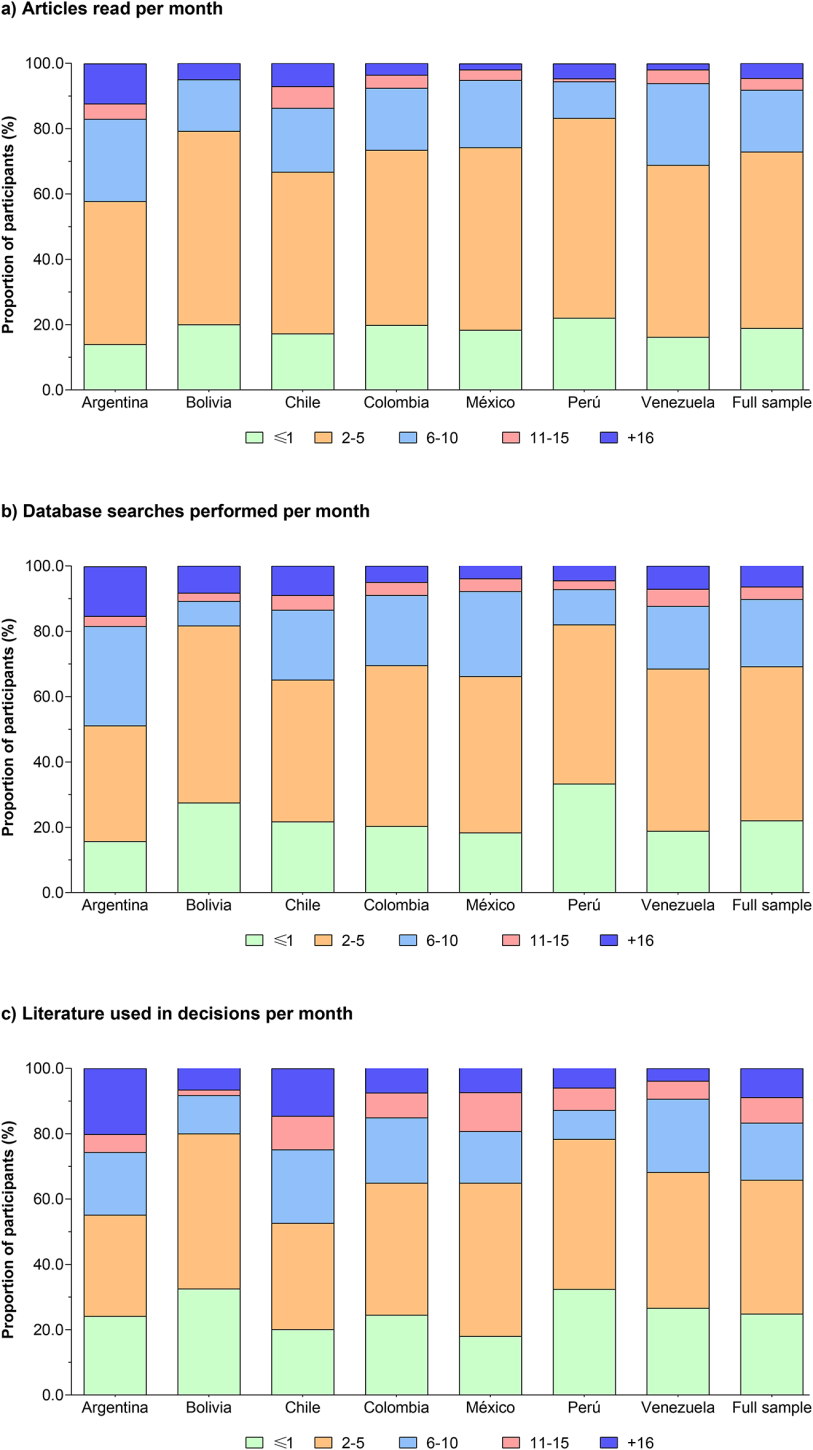
Self-reported access to and availability of literature.

Four out of five respondents (80.9%) reported having access to professional journals in paper form. 74% of the respondents contended that clinical guidelines relevant to their practice areas were available, and only 25.7% stated that they had access to those guidelines online. More respondents stated they had access to relevant databases and the Internet at home (86%) but not at work (63.4%), Fig. 5. Within the sub-group item “personal use and understanding of clinical practice guidelines,” most of the PTs access and know the practice guidelines and incorporate them into patient preferences (68.6%, and 80.9%, respectively).

**Fig. 6:**
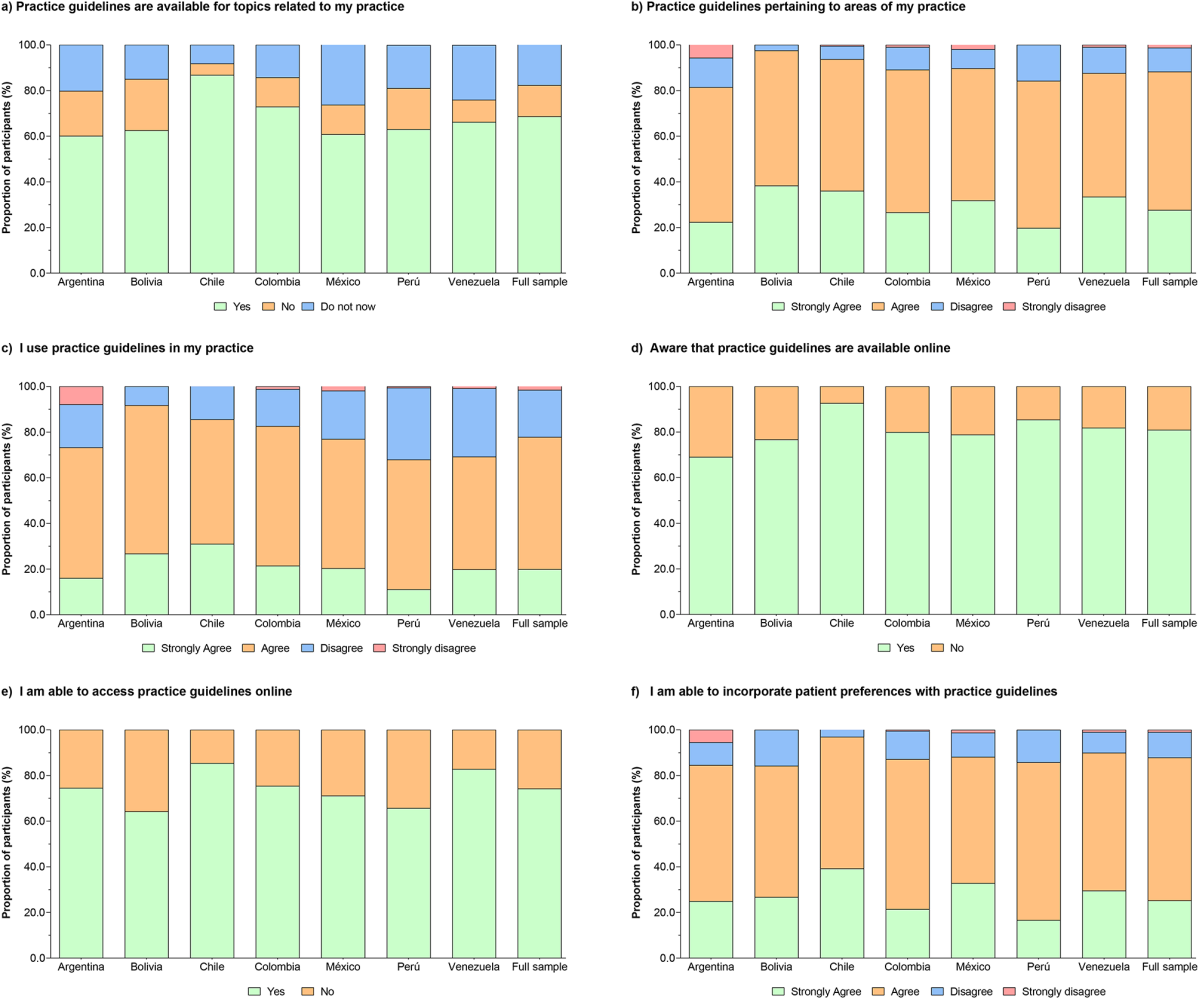
Personal use and understanding of clinical practice guidelines.

Figure 6 Male gender, having “participated in continuing education courses,” and being a “clinical instructor” were significantly associated with using the guidelines in practice. Furthermore, those who had access to guidelines and incorporated them into patients’ preferences stated they agreed among PTs belonging to the “participated in continuing education courses” (OR = 3.1, 95% CI 2.5–3.8), belonging to the “professional membership register” (OR = 2.7, 95% CI 2.0–3.5), and belonging to the “clinical instructors” (OR = 2.6, 95% CI 1.9–3.6), in comparison with their peers (*P* < 0.001). Table S5 summarizes the sub-group logistic regression information. Those therapists in the “university setting,” who had “participated in continuing education courses,” or belonged to “clinical instructors” were more likely to agree to times “access to online databases in practice” in comparison with their peers (*P* < 0.001). Table S6 summarizes the sub-group logistic regression information.

### Self-reported ranking of barriers to evidence-based practice

**Fig. 7:**
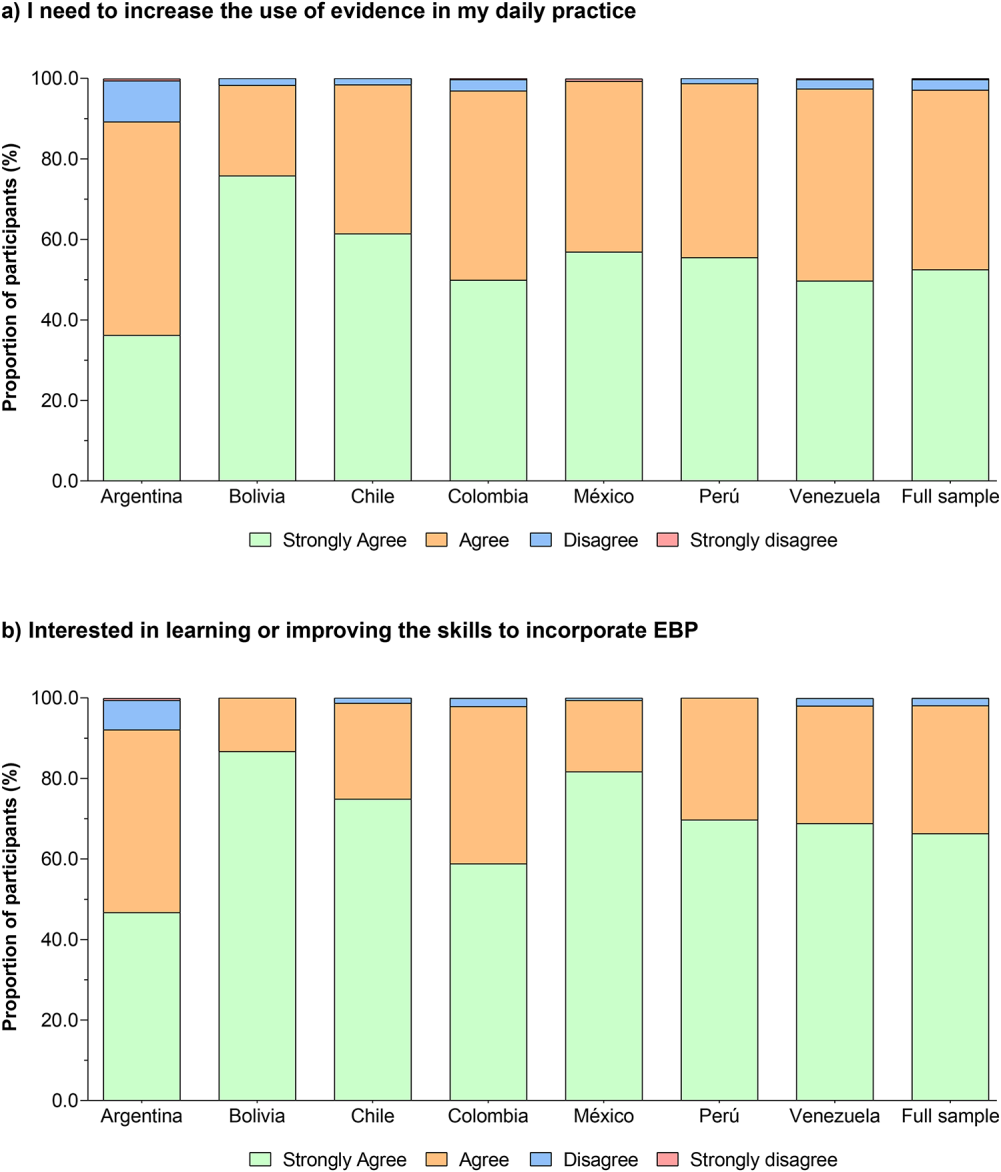
Self-reported ranking of barriers to evidence-based practice.

53% of respondents indicated that insufficient time was the most important barrier to the use of EBP. Lack of information resources was chosen as the second most important barrier by 20.8% of respondents, and approximately 13.6% rated a lack of research skills as the third most important barrier, Fig. 7. PTs were more likely to report working 41–40 h hours/week as a barrier to the use of EBP than “lack of information resources” and “lack of research skills” in comparison with their peers (*P* < 0.001). Table S7 summarizes the sub-group logistic regression information.

## Discussion

The overall results showed that Latin American PTs who responded to our survey viewed EBP as an essential component of practice. Respondents thought research literature was useful in day-to-day practice and indicated that EBP improved the quality of their patient care and assists in decision making. Respondents also expressed an interest in further developing EBP skills. These findings were consistent with results in studies of nurses, general practitioners, physiotherapists, occupational therapists, speech-language therapists and complementary therapists^1–6,18–24^. This aligns with previous studies, such as that of Ramírez-Vélez et al., which found a high proportion of young, female physiotherapists in Colombia^8^. One possible explanation could be that the greater emphasis on EBP during their professional training with more exposure on how to integrate evidence in clinical decision-making.

Although 61.1% held only a bachelor’s degree, 66.7% expressed interest in advanced degrees. This mirrors the high participation in continuing education courses and specialist certifications, consistent with U.S. findings on the importance of continuing education^19^. 21% belonged to practice-oriented organizations, aiding access to resources. Most worked over 40 h a week, primarily treating orthopedic and cardiovascular/respiratory conditions (47.2% and 24.4%). This workload and clinical focus agree with the findings of a Saudi Arabian study^20^.

Most worked in urban settings and small clinics, which present challenges and opportunities like multifunctionality and closer patient relationships. However, research and teaching were low priorities, with less than 25% of time dedicated to these activities, indicating a need for greater institutional support, as highlighted in Spanish studies^19^. The moderate correlation between age and years since licensure (rs = 0.72) suggests that younger physiotherapists are new to practice, emphasizing the need for support and mentoring, a point also noted in the literature^1^.

The high percentage of PTs who consider EBP necessary and beneficial reflects a global trend towards the acceptance of EBP as a standard practice. This finding is consistent with previous studies, such as those by Jette et al.^3^ and Scurlock-Evans et al.^18^, which also found positive attitudes towards EBP. However, the perception that there is a lack of solid evidence to support certain practices suggests a need for improved training and access to updated research resources. Alsaadi et al.^2^ also noted that the perception of a lack of evidence can discourage physical therapists from implementing EBP in their daily practice.

Although most participants feel confident in their search skills and knowledge of databases, fewer have strong skills in critically appraising research. This finding is consistent with previous studies that also reported a gap in comprehensive EBP training^21,22^. Participation in continuing education is associated with greater confidence in EBP skills, highlighting the importance of ongoing professional development opportunities^23^.

The varied understanding of terms such as “odds ratio” and “publication bias” suggests a need for educational interventions focused on statistical literacy and the interpretation of research findings^24^. Male PTs and those involved in continuing education or professional memberships showed greater knowledge of these terms, indicating that professional engagement can improve EBP literacy^25^. Most PTs report reading and using 2–5 articles per month for clinical decision-making. This shows a positive engagement with the literature, although it could be enhanced with easier and quicker access to resources in the workplace^2,26^.

Access to professional journals and clinical guidelines is generally high among PTs, but there is a notable discrepancy between access at home and at work. This limitation could affect the application of EBP in clinical settings where quick access to information is crucial^26^. The strong association between participation in continuing education and better access to resources underscores the importance of institutional support for professional development^23^.

The results of our study align with previous findings indicating a generally positive attitude toward EBP among PTs, despite significant barriers. Jette et al.^3^ found that physical therapists in the United States recognize the importance of EBP but face obstacles such as a lack of time and resources. Similarly, Scurlock-Evans et al.^18^ identified barriers in the United Kingdom, including a lack of skills and misconceptions about EBP. Ramírez-Vélez et al.^8^ found that Colombian PTs have positive attitudes towards EBP but face barriers such as a lack of research skills, consistent with our findings. This suggests that despite geographical and cultural differences, the challenges to implementing EBP are similar across various regions. Additionally, Alsaadi’s study in Saudi Arabia highlighted similar barriers, underscoring the global nature of these challenges^2^.

Addressing these barriers requires a multifaceted approach. Enhancing access to research databases and professional journals within clinical settings can facilitate the application of EBP. Implementing time management strategies and providing dedicated time for professional development during work hours could alleviate the time constraints faced by PTs^27,28^. Furthermore, incorporating comprehensive EBP training into undergraduate and postgraduate curricula, as well as offering regular workshops and courses on EBP skills, can improve the proficiency of PTs in interpreting and applying research evidence^29^. Therefore, we recommend that if further studies are conducted that they identify barriers particular to the use of clinical guidelines in PTs practice. Institutional support in the form of mentorship programs for younger PTs and incentives for engaging in EBP activities can also promote a culture of continuous learning and evidence-based practice^8^.

## Limitations

Among the limitations, we can mention that this is a cross-sectional study; therefore, it is not possible to determine causality but only to establish associations. In addition, due to the online nature of the questionnaire, professionals with connectivity issues who live in rural areas, individuals with uncorrected visual impairments, and/or those who are unfamiliar with social media may have been excluded from the study. Most respondents stated that they read research literature, but the survey instrument did not capture information regarding the quality of the research literature. As such, it is unclear if the articles were sound scientific papers or opinion-based commentaries. The other side, our sample size is reasonable and the generalisability of our findings is partially supported by the similarities in demographic characteristics between our survey respondents and demographic material made available by the Latin American PTs. Considered together, this suggests that caution is needed in determining whether the views of our sample towards EBP would be representative of all Latin American PTs.

## Conclusion

This study found that most Latin American PTs regularly read relevant literature and apply this knowledge in their clinical practice. This is more likely among those with less than five years of experience and those who see 4–5 patients per day. In addition, a lack of information resources and research skills were significant barriers. Our findings highlighting the need for improved access to resources and training in research skills as key factors to promote EBP in clinical decision-making. Future research should address these barriers and improve understanding of complex terms to promote more robust and effective evidence-based practice.

## Electronic supplementary material

Below is the link to the electronic supplementary material.

Supplementary Material 1

## Data Availability

The summary dataset used and or analyzed during the current study are available from the corresponding author on a reasonable request.
