# Supplementary material for: A survey of beliefs, attitudes, knowledge, and behaviors about evidence-based practice in physical therapists of Latin America: a cross-sectional study
Source: Sci Rep. 2024 Nov 9;14:27404. doi: 10.1038/s41598-024-78703-w (PMC11550410; doi:10.1038/s41598-024-78703-w)
Supplement: Supplementary file 1 — Supplementary Material 1 [file 41598_2024_78703_MOESM1_ESM.pdf]

## SUPPLEMENTARY MATERIALS

### eMethods

### *Data analyses*

Using a similar strategy to that of Jette et al. (1) and Ferreira et al. (2), for items with a four-point Likert scale, the "Strongly Agree" and "Agree" categories were combined, as well as the "Strongly Disagree" and "Disagree" categories, so that responses fell into one of two categories: "Agree" or "Disagree". For the items with a "Yes/No/Do Not Know" choice set, the "Do Not Know" category was combined with the "No" category, based on the belief that a lack of knowledge regarding EBP topics is similar to not performing EBP. For items categorized by the number of times, the lowest categories (≤1 and 2–5) were distinguished from the higher categories (6–10, 11–15, and ≥16), and combined into "Poor" for the lower values and "Good" for the high values. For items that were designed to examine the degree of understanding of research terms, the "Understand Completely" and "Understand Somewhat" categories were combined so that a 2-category response was obtained: "Understand Completely" and "Understand at Least Somewhat plus Do Not Understand". Lastly, in the barriers item, the PTs' choices were collapsed into "Present" (if the PT chooses 1$^{st}$, 2$^{nd}$, or 3$^{rd}$) or "Absent" (no barrier choice). After item categories were collapsed, logistic regression tests were used to assess the association between the demographics and research-related items (attitudes and beliefs about EBP; interest in and motivation to engage in EBP; educational background, knowledge, and skills related to accessing and interpreting information; the level of attention to and use of the literature; access to and availability of information to promote EBP; and perceived barriers to using EBP). An $\alpha$ of 0.05 was used to define whether a model needed to be reported. Odds ratios (ORs) and their 95% CIs were determined for each level of the independent variables controlling for the country. CIs including 1.0 were considered not statistically significant

**Table S1**. Association between PTs' characteristics and their attitudes, beliefs, interest in and motivation to about/engage EBP.

| Attitude or belief (Agree) | Factor – Level | Odds Ratio (95% CI) | ***Model P*** | ***Model*** $R^{2}$ [a] |
|---|---|---|---|---|
| EBP is Necessary to Practice | | | | |
| | *Years since licensure* | | *0.002* | *0.117* |
| | <5 y | 4.599 (1.919–11.021) | | |
| | 5–10 y | 4.025 (1.687–9.601) | | |
| | 11–15 y | 2.729 (1.013–7.352) | | |
| | >15 y | Reference[b] | | |
| EBP improves the quality of patient care | | | | |
| | *Age* | | *<0.001* | *0.032* |
| | 20–29 y | 2.188 (1.298–3.687) | | |
| | 30–39y | 3.169 (1.723–5.831) | | |
| | 40–49 y | 0.912 (0.522–1.595) | | |
| | >50 y | Reference[b] | | |
| | *Years since licensure* | | *0.020* | *0.050* |
| | <5 y | 2.717 (1.841–4.010) | | |
| | 5–10 y | 5.430 (3.106–9.495) | | |
| | 11–15 y | 2.415 (1.395–4.181) | | |
| | >15 y | Reference[b] | | |
| Reimbursement will increase with use of EBP | | | | |
| | *Years since licensure* | | *<0.001* | *0.081* |
| | <5 y | 2.684 (2.253–3.197) | | |
| | 5–10 y | 1.920 (1.587–2.323) | | |
| | 11–15 y | 1.650 (1.313–2.073) | | |
| | >15 y | Reference[b] | | |
| EBP doesn't account for practice settings limitations | | | | |
| | *Years since licensure* | | *<0.001* | *0.024* |
| | <5 y | 2.023 (1.703–2.402) | | |
| | 5–10 y | 1.598 (1.323–1.932) | | |
| | 11–15 y | 1.625 (1.292–2.045) | | |
| | >15 y | Reference[b] | | |
| | *Working hours* | | *<0.001* | *0.011* |
| | < 20 | 1.572 (1.300–1.902) | | |
| | 20–30 | 1.313 (1.093–1.577) | | |
| | 31–40 | 1.407 (1.186–1.670) | | |
| | > 40 | Reference[b] | | |
| Evidence is Lacking to Support Interventions | | | | |
| | *Working hours* | | *<0.001* | *0.049* |
| | < 20 h | 2.383 (1.977–2.873) | | |
| | 20–30 h | 2.412 (2.010–2.895) | | |
| | 31–40 h | 2.302 (1.941–2.730) | | |
| | > 40 h | Reference[b] | | |
| | *Years since licensure* | | *<0.001* | *0.036* |
| | <5 y | 2.172 (1.822–2.588) | | |
| | 5–10 y | 2.218 (1.829–2.690) | | |
| | 11–15 y | 1.224 (0.968–1.549) | | |
| | >15 y | Reference[b] | | |
| | *Age* | | *<0.001* | *0.016* |
| | 20–29 y | 1.797 (1.404–2.300) | | |
| | 30–39y | 1.301 (1.004–1.687) | | |
| | 40–49 y | 1.093 (0.821–1.454) | | |
| | >50 y | Reference[b] | | |
| EBP helps decision making | | | | |
| | *Years since licensure* | | *<0.001* | *0.059* |
| | <5 y | 5.448 (3.473–8.545) | | |
| | 5–10 y | 3.984 (2.515–6.313) | | |

| | | | | |
|---|---|---|---|---|
| | 11–15 y | 4.527 (2.374–8.635) | | |
| | >15 y | Reference[b] | | |
| | *Age* | | *<0.001* | *0.059* |
| | 20–29 y | 4.098 (2.561–6.558) | | |
| | 30–39y | 5.601 (3.169–9.899) | | |
| | 40–49 y | 1.607 (0.964–2.680) | | |
| | >50 y | Reference[b] | | |
| Necessity to increase evidence use in practice | | | | |
| | *Academic degree* | | *<0.001* | *0.053* |
| | Undergraduate | 3.664 (1.395–9.624) | | |
| | Specialization | 5.238 (1.781–15.407) | | |
| | Master | 3.511 (1.221–10.101) | | |
| | Doctorate | Reference[b] | | |
| | *Patients day* | | | |
| | <5 | 1.912 (1.145–3.192) | *0.007* | *0.060* |
| | 5–10 | 2.270 (1.379–3.734) | | |
| | 11–15 | 2.288 (1.307–4.006) | | |
| | >15 | Reference[b] | | |
| Learning/Improving skills interest to incorporate EBP | | | | |
| | *Years since licensure* | | *<0.001* | *0.146* |
| | <5 y | 5.138 (2.824–9.348) | | |
| | 5–10 y | 10.917 (4.617–25.815) | | |
| | 11–15 y | 3.081 (1.528–6.211) | | |
| | >15 y | Reference[b] | | |
| | Professional membership register | | | |
| | Yes | 2.225 (1.158–4.276) | *<0.001* | *0.073* |
| | No | Reference[b] | | |
| | *Patients day* | | *<0.001* | *0.081* |
| | <5 | 1.811 (0.972–3.374) | | |
| | 5–10 | 2.818 (1.484–5.349) | | |
| | 11–15 | 2.318 (1.179–4.558) | | |
| | >15 | Reference[b] | | |

[a] Nagelkerke $R^2$; [b] In logistic regression, one level of the independent variable serve as reference against which the odds of the other levels occurring are determined. 95% CI=95% confidence interval. Controlling for country.

**Table S2.** Association between PTs' characteristics and their educational background, knowledge and skills related to accessing and interpreting information.

| Education, Skill or Knowledge (Agree) | Factor – Level | Odds Ratio (95% CI) | *P* | $R^{2\,a}$ |
|---|---|---|---|---|
| Learned foundations in academic program | | | | |
| | *Age* | | <0.001 | 0.056 |
| | 20–29 y | 3.328 (2.493–4.443) | | |
| | 30–39 y | 1.987 (1.477–2.674) | | |
| | 40–49 y | 0.978 (0.718–1.331) | | |
| | >50 y | Reference[b] | | |
| | *Years since licensure* | | <0.001 | 0.113 |
| | <5 y | 4.349 (3.518–5.375) | | |
| | 5–10 y | 5.428 (4.180–7.049) | | |
| | 11–15 y | 4.639 (3.357–6.411) | | |
| | >15 y | Reference[b] | | |
| | *Participated in continuing education courses* | | <0.001 | 0.007 |
| | Yes | 1.504 (1.239–1.827) | | |
| | No | Reference[b] | | |
| | *Professional membership register* | | | |
| | Yes | 1.305 (1.042–1.633) | <0.001 | 0.003 |
| | No | Reference[b] | | |
| Familiar with online databases | | | | |
| | *Age* | | 0.002 | 0.059 |
| | 20–29 y | 2.405 (1.779–3.251) | | |
| | 30–39 y | 1.566 (1.147–2.139) | | |
| | 40–49 y | 0.624 (0.454–0.857) | | |
| | >50 y | Reference[b] | | |
| | *Years since licensure* | | <0.001 | 0.093 |
| | <5 y | 4.178 (3.371–5.179) | | |
| | 5–10 y | 4.574 (3.540–5.910) | | |
| | 11–15 y | 2.543 (1.922–3.364) | | |
| | >15 y | Reference[b] | | |
| | *Participated in continuing education courses* | | <0.001 | 0.065 |
| | Yes | 3.248 (2.709–3.894) | | |
| | No | Reference[b] | | |
| | *Professional membership register* | | | |
| | Yes | 3.590 (2.663–4.840) | <0.001 | 0.040 |
| | No | Reference[b] | | |
| | *Clinical Instructor* | | <0.001 | 0.030 |
| | Yes | 2.544 (2.006–3.226) | | |
| | No | Reference[b] | | |
| Formal training in critical appraisal | | | | |
| | *Age* | | <0.001 | 0.062 |
| | 20–29 y | 2.279 (1.785–2.910) | | |
| | 30–39 y | 1.460 (1.132–1.884) | | |
| | 40–49 y | 0.715 (0.543–0.942) | | |
| | >50 y | Reference[b] | | |
| | *Years since licensure* | | <0.001 | 0.104 |
| | <5 y | 3.223 (2.701–3.844) | | |
| | 5–10 y | 3.867 (3.149–4.749) | | |
| | 11–15 y | 1.159 (0.927–1.450) | | |
| | >15 y | Reference[b] | | |
| | *Academic Degree* | | <0.001 | 0.020 |
| | Undergraduate | 0.230 (0.098–0.542) | | |
| | Specialization | 0.236 (0.099–0.561) | | |
| | Master | 0.357 (0.149–0.852) | | |
| | Doctorate | Reference[b] | | |

| | | | | |
|---|---|---|---|---|
| | *Participated in continuing education courses* | | *<0.001* | *0.025* |
| | Yes | 1.732 (1.484–2.021) | | |
| | No | Reference[b] | | |
| | *Professional membership register* | | | |
| | Yes | 1.693 (1.419–2.020) | *<0.001* | *0.021* |
| | No | Reference[b] | | |
| | *Clinical Instructor* | | *<0.001* | *0.032* |
| | Yes | 1.952 (1.658–2.297) | | |
| | No | Reference[b] | | |
| Formal training in search strategies | | | | |
| | *Age* | | *<0.001* | *0.045* |
| | 20–29 y | 1.846 (1.420–2.401) | | |
| | 30–39 y | 1.255 (0.955–1.649) | | |
| | 40–49 y | 0.599 (0.448–1.299) | | |
| | >50 y | Reference[b] | | |
| | *Years since licensure* | | *<0.001* | *0.089* |
| | <5 y | 3.202 (2.656–3.860) | | |
| | 5–10 y | 3.419 (2.755–4.242) | | |
| | 11–15 y | 1.034 (0.824–1.297) | | |
| | >15 y | Reference[b] | | |
| | *Participated in continuing education courses* | | *<0.001* | *0.012* |
| | Yes | 1.628 (1.383–1.916) | | |
| | No | Reference[b] | | |
| | *Professional membership register* | | | |
| | Yes | 2.540 (2.058–3.135) | *<0.001* | *0.032* |
| | No | Reference[b] | | |
| | *Clinical Instructor* | | *<0.001* | *0.030* |
| | Yes | 2.226 (1.853–2.674) | | |
| | No | Reference[b] | | |
| | *Working hours* | | *<0.001* | *0.046* |
| | < 20 | 1.609 (1.313–1.971) | | |
| | 20–30 | 1.670 (1.367–2.040) | | |
| | 31–40 | 2.305 (1.895–2.805) | | |
| | > 40 | Reference[b] | | |
| | *Patients day* | | *<0.001* | *0.095* |
| | 1–5 | 3.607 (2.939–4.426) | | |
| | 6–10 | 4.538 (3.683–5.591) | | |
| | 11–15 | 4.389 (3.481–5.535) | | |
| | > 15 | Reference[b] | | |
| Confident in critical appraisal skills | | | | |
| | *Age* | | *<0.001* | *0.062* |
| | 20–29 y | 2.279 (1.785–2.910) | | |
| | 30–39 y | 1.460 (1.132–1.884) | | |
| | 40–49 y | 0.715 (0.543–1.042) | | |
| | >50 y | Reference[b] | | |
| | *Years since licensure* | | *<0.001* | *0.104* |
| | <5 y | 3.223 (2.701–3.844) | | |
| | 5–10 y | 3.867 (3.149–4.749) | | |
| | 11–15 y | 1.159 (0.927–1.450) | | |
| | >15 y | Reference[b] | | |
| | *Participated in continuing education courses* | | *<0.001* | *0.025* |
| | Yes | 1.732 (1.484–2.021) | | |
| | No | Reference[b] | | |

| | | | | |
|---|---|---|---|---|
| | *Professional membership register* | | | |
| | Yes | 1.693 (1.419–2.020) | *<0.001* | *0.021* |
| | No | Reference[b] | | |
| | *Clinical Instructor* | | *<0.001* | *0.032* |
| | Yes | 1.952 (1.658–2.297) | | |
| | No | Reference[b] | | |
| | *Working hours* | | *<0.001* | *0.024* |
| | < 20 | 1.766 (1.452–2.147) | | |
| | 20–30 | 1.627 (1.344–1.969) | | |
| | 31–40 | 1.449 (1.217–1.725) | | |
| | > 40 | Reference[b] | | |
| | *Patients day* | | *<0.001* | *0.074* |
| | 1–5 | 3.183 (2.609–3.882) | | |
| | 6–10 | 3.538 (2.900–4.317) | | |
| | 11–15 | 3.041 (2.452–3.771) | | |
| | > 15 | Reference[b] | | |
| Confident in search skills | | | | |
| | *Years since licensure* | | *<0.001* | *0.038* |
| | <5 y | 1.417 (1.116–1.799) | | |
| | 5–10 y | 3.056 (2.215–4.217) | | |
| | 11–15 y | 3.783 (2.411–5.936) | | |
| | >15 y | Reference[b] | | |
| | *Participated in continuing education courses* | | *<0.001* | *0.104* |
| | Yes | 4.838 (3.909–5.988) | | |
| | No | Reference[b] | | |
| | *Professional membership register* | | | |
| | Yes | 5.483 (3.538–8.499) | *<0.001* | *0.048* |
| | No | Reference[b] | | |
| | *Clinical Instructor* | | *<0.001* | *0.018* |
| | Yes | 2.165 (1.639–2.860) | | |
| | No | Reference[b] | | |

[a] Nagelkerke $R^2$; [b] In logistic regression, one level of the independent variable serve as reference against which the odds of the other levels occurring are determined. 95% CI=95% confidence interval. Controlling for country.

**Table S3.** Factors associated with understanding of specific terms.

| The therapists' self–evaluated knowledge of terms associated with EBP (largely understood) | Factor – Level | Odds Ratio (95% CI) | *P* | $R^2$ [a] |
|---|---|---|---|---|
| Relative risk | | | | |
| | *Sex* | | <0.001 | 0.036 |
| | Male | 1.979 (1.729–2.266) | | |
| | Female | Reference[b] | | |
| | *Participated in continuing education courses* | | <0.001 | 0.058 |
| | Yes | 2.752 (2.350–3.222) | | |
| | No | Reference[b] | | |
| | *Professional membership register* | | | |
| | Yes | 2.635 (2.237–3.104) | <0.001 | 0.050 |
| | No | Reference[b] | | |
| | *Clinical Instructor* | | <0.001 | 0.034 |
| | Yes | 2.049 (1.772–2.370) | | |
| | No | Reference[b] | | |
| Absolute risk | | | | |
| | *Sex* | | <0.001 | 0.019 |
| | Male | 1.615 (1.410–1.851) | | |
| | Female | Reference[b] | | |
| | *Participated in continuing education courses* | | <0.001 | 0.026 |
| | Yes | 1.873 (1.610–2.178) | | |
| | No | Reference[b] | | |
| | *Professional membership register* | | | |
| | Yes | 2.297 (1.944–2.714) | <0.001 | 0.037 |
| | No | Reference[b] | | |
| | *Clinical Instructor* | | <0.001 | 0.017 |
| | Yes | 1.614 (1.394–1.868) | | |
| | No | Reference[b] | | |
| Systematic review | | | | |
| | *Sex* | | <0.001 | 0.019 |
| | Male | 1.822 (1.571–2.114) | | |
| | Female | Reference[b] | | |
| | *Participated in continuing education courses* | | <0.001 | 0.040 |
| | Yes | 2.075 (1.779–2.421) | | |
| | No | Reference[b] | | |
| | *Professional membership register* | | <0.001 | 0.057 |
| | Yes | 2.957 (2.425–3.606) | | |
| | No | Reference[b] | | |
| | *Clinical Instructor* | | <0.001 | 0.047 |
| | Yes | 2.306 (1.949–2.728) | | |
| | No | Reference[b] | | |
| Odds ratio | | | | |
| | *Sex* | | <0.001 | 0.068 |
| | Male | 3.055 (2.556–3.652) | | |
| | Female | Reference[b] | | |
| | *Participated in continuing education courses* | | <0.001 | 0.023 |
| | Yes | 2.329 (1.800–3.014) | | |
| | No | Reference[b] | | |

| | | | | |
|---|---|---|---|---|
| | *Professional membership register* | | *<0.001* | *0.056* |
| | Yes | 3.019 (2.503–3.642) | | |
| | No | Reference[b] | | |
| | *Clinical Instructor* | | *<0.001* | *0.079* |
| | Yes | 3.454 (2.887–4.132) | | |
| | No | Reference[b] | | |
| Meta–analysis | | | | |
| | *Sex* | | *<0.001* | *0.055* |
| | Male | 2.280 (1.992–2.610) | | |
| | Female | Reference[b] | | |
| | *Participated in continuing education courses* | | *<0.001* | *0.048* |
| | Yes | 2.423 (2.068–2.839) | | |
| | No | Reference[b] | | |
| | *Professional membership register* | | *<0.001* | *0.091* |
| | Yes | 3.740 (3.160–4.427) | | |
| | No | Reference[b] | | |
| | *Clinical Instructor* | | *<0.001* | *0.061* |
| | Yes | 2.575 (2.224–2.982) | | |
| | No | Reference[b] | | |
| Confidence interval | | | | |
| | *Sex* | | *<0.001* | *0.040* |
| | Male | 2.059 (1.802–2.354) | | |
| | Female | Reference[b] | | |
| | *Participated in continuing education courses* | | *<0.001* | *0.043* |
| | Yes | 2.434 (2.070–2.861) | | |
| | No | Reference[b] | | |
| | *Professional membership register* | | *<0.001* | *0.069* |
| | Yes | 3.125 (2.660–3.672) | | |
| | No | Reference[b] | | |
| | *Clinical Instructor* | | *<0.001* | *0.055* |
| | Yes | 2.510 (2.172–2.900) | | |
| | No | Reference[b] | | |
| Heterogeneity | | | | |
| | *Sex* | | *<0.001* | *0.056* |
| | Male | 1.994 (1.743–2.281) | | |
| | Female | Reference[b] | | |
| | *Participated in continuing education courses* | | *<0.001* | *0.073* |
| | Yes | 2.697 (2.293–3.173) | | |
| | No | Reference[b] | | |
| | *Professional membership register* | | | |
| | Yes | 2.811 (2.390–3.307) | *<0.001* | *0.076* |
| | No | Reference[b] | | |
| | *Clinical Instructor* | | *<0.001* | *0.058* |
| | Yes | 2.138 (1.850–2.472) | | |
| | No | Reference[b] | | |
| Publication bias | | | | |
| | *Sex* | | *<0.001* | *0.045* |
| | Male | 2.158 (1.887–2.467) | | |
| | Female | Reference[b] | | |
| | *Participated in continuing education courses* | | *<0.001* | *0.049* |

| | | | |
|---|---|---|---|
| Yes | 2.593 (2.202–3.053) | | |
| No | Reference[b] | | |
| *Professional membership register* | | <0.001 | 0.094 |
| Yes | 3.883 (3.290–4.582) | | |
| No | Reference[b] | | |
| *Clinical Instructor* | | <0.001 | 0.068 |
| Yes | 2.792 (2.413–3.229) | | |
| No | Reference[b] | | |

[a] Nagelkerke $R^2$; [b] In logistic regression, one level of the independent variable serve as reference against which the odds of the other levels occurring are determined. 95% CI=95% confidence interval. Controlling for country.

**Table S4**. Association between PTs' characteristics and their attention to and use of the literature.

| Attention an use of literature (Good) | Factor – Level | Odds Ratio (95% CI) | *Model P* | *Model $R^{2}$* [a] |
|---|---|---|---|---|
| Articles read per month | | | | |
| | *Age* | | *<0.001* | *0.061* |
| | 20–29 y | 3.492 (2.720–4.483) | | |
| | 30–39y | 2.064 (1.593–2.674) | | |
| | 40–49 y | 1.522 (1.148–2.018) | | |
| | >50 y | Reference[b] | | |
| | *Years since licensure* | | *<0.001* | *0.096* |
| | <5 y | 3.752 (3.109–4.527) | | |
| | 5–10 y | 3.069 (2.500–3.768) | | |
| | 11–15 y | 1.471 (1.169–1.852) | | |
| | >15 y | Reference[b] | | |
| | *Working hours* | | *<0.001* | *0.074* |
| | < 20 | 2.396 (1.947–2.948) | | |
| | 20–30 | 2.503 (2.045–3.064) | | |
| | 31–40 | 2.941 (2.427–3.565) | | |
| | > 40 | Reference[b] | | |
| | *Patients day* | | *<0.001* | *0.073* |
| | <5 | 2.581 (2.112–3.154) | | |
| | 5–10 | 3.252 (2.657–3.979) | | |
| | 11–15 | 3.156 (2.402–4.076) | | |
| | >15 | Reference[b] | | |
| Literature used in decisions per month | | | | |
| | *Sex* | | *0.010* | *0.015* |
| | Male | 1.583 (1.372–1.827) | | |
| | Female | Reference[b] | | |
| | *Professional membership register* | | | |
| | Yes | 1.209 (1.023–1.429) | *<0.001* | *0.002* |
| | No | Reference[b] | | |

[a] Nagelkerke $R^2$; [b] In logistic regression, one level of the independent variable serve as reference against which the odds of the other levels occurring are determined. 95% CI=95% confidence interval. Controlling for country.

**Table S5.** Association between PTs' characteristics and their access to practical guidelines.

| **Access to Practical Guidelines** | **Factor – Level** | **Odds Ratio (95% CI)** | ***Model P*** | ***Model $R^{2}$*** [a] |
|---|---|---|---|---|
| Guidelines use in practice (Agree) | | | | |
| | *Participated in continuing education courses* | | *<0.001* | *0.082* |
| | Yes | 2.986 (2.532–3.522) | | |
| | No | Reference[b] | | |
| | *Clinical Instructor* | | *<0.001* | *0.057* |
| | Yes | 2.604 (2.123–3.195) | | |
| | No | Reference[b] | | |
| | *Sex* | | *<0.001* | *0.031* |
| | Male | 1.554 (1.313–1.838) | | |
| | Female | Reference[b] | | |
| Guidelines patients' preferences incorporation (Agree) | | | | |
| | *Participated in continuing education courses* | | *<0.001* | *0.082* |
| | Yes | 3.133 (2.566–3.825) | | |
| | No | Reference[b] | | |
| | *Clinical Instructor* | | *<0.001* | *0.030* |
| | Yes | 2.708 (2.057–3.564) | | |
| | No | Reference[b] | | |
| | *Professional membership register* | | | |
| | Yes | 2.681 (1.971–3.647) | *<0.001* | *0.024* |
| | No | Reference[b] | | |

[a] Nagelkerke $R^2$; [b] In logistic regression, one level of the independent variable serve as reference against which the odds of the other levels occurring are determined. 95% CI=95% confidence interval. Controlling for country.

**Table S6.** Association between PTs' characteristics and their access to and availability of information to promote EBP.

| Access and Availably of Information | Factor – Level | Odds Ratio (95% CI) | *Model P* | *Model $R^{2}$* [a] |
|---|---|---|---|---|
| Access to relevant databases in practice (Agree) | | | | |
| | *Participated in continuing education courses* | | <0.001 | 0.082 |
| | Yes | 2.986 (2.532–3.522) | | |
| | No | Reference | | |
| | *Clinical Instructor* | | <0.001 | 0.057 |
| | Yes | 2.604 (2.123–3.195) | | |
| | No | Reference[b] | | |
| | *Professional membership register* | | | |
| | Yes | 2.254 (1.803–2.818) | <0.001 | 0.043 |
| | No | Reference[b] | | |
| | *Sex* | | | |
| | Male | 1.554 (1.313–1.838) | <0.001 | 0.031 |
| | Female | Reference[b] | | |
| | *Working hours* | | <0.001 | 0.046 |
| | < 20 | 1.551 (1.257–1.915) | | |
| | 20–30 | 2.296 (1.827–2.885) | | |
| | 31–40 | 1.847 (1.511–2.257) | | |
| | > 40 | Reference[b] | | |
| | *Patients day* | | <0.001 | 0.061 |
| | 1–5 | 1.946 (1.574 2.404) | | |
| | 6–10 | 2.980 (2.384 3.726) | | |
| | 11–15 | 2.680 (2.097 3.425) | | |
| | > 15 | Reference[b] | | |
| | *Type of facility* | | 0.008 | 0.036 |
| | Acute care hospital | 1.338 (0.941–1.904) | | |
| | Acute rehabilitation | 1.568 (1.029–2.390) | | |
| | Subacute rehabilitation | 1.312 (0.828–2.078) | | |
| | Skilled nursing facility | 1.616 (1.071–2.436) | | |
| | Private outpatient clinic | 1.141 (0.842–1.546) | | |
| | Facility–based outpatient clinic | 1.305 (0.920–1.851) | | |
| | Home care | 1.402 (1.006–1.955) | | |
| | School system | 1.225 (0.585–2.566) | | |
| | University | 2.832 (1.884–4.254) | | |
| | Other | Reference[b] | | |
| | Condition for majority of patients treated | | 0.003 | 0.029 |
| | Orthopedic | 1.630 (0.816–3.255) | | |
| | Neurological | 1.838 (0.890–3.796) | | |
| | Cardiovascular/respiratory | 2.405 (1.192–4.851) | | |
| | Pediatric/neonatal | 2.363 (0.957–5.836) | | |
| | Geriatric | 1.556 (0.423–5.724) | | |
| | Sports rehabilitation | 1.314 (0.625–2.759) | | |
| | Others | 2.011 (0.956–4.226) | | |
| | No patient care | Reference[b] | | |

[a] Nagelkerke $R^2$; [b] In logistic regression, one level of the independent variable serve as reference against which the odds of the other levels occurring are determined. 95% CI=95% confidence interval. Controlling for country.

**Table S7**. Association Between PTs' Characteristics and Their Perceived Barriers.

| Access and Availably of Information | Factor – Level | Odds Ratio (95% CI) | *Model P* | *Model $R^{2}$* [a] |
|---|---|---|---|---|
| Insufficient time | | | | |
| | *Years of License* | | <0.001 | 0.048 |
| | < 5 | 0.473 (0.371–0.603) | | |
| | 5–10 | 1.028 (0.795–1.329) | | |
| | 11–15 | 1.025 (0.773–1.359) | | |
| | > 15 | Reference[b] | | |
| Lack of information resources | | | | |
| | *Working hours* | | <0.001 | 0.052 |
| | < 20 | 2.258 (1.715–2.973) | | |
| | 20–30 | 2.218 (1.659–2.966) | | |
| | 31–40 | 3.260 (2.470–4.304) | | |
| | > 40 | Reference[b] | | |
| | *Patients day* | | <0.001 | 0.026 |
| | 1–5 | 1.812 (1.328–2.473) | | |
| | 6–10 | 1.607 (1.175–2.199) | | |
| | 11–15 | 1.936 (1.395–2.688) | | |
| | > 15 | Reference[b] | | |
| | *Clinical Instructor* | | <0.001 | 0.037 |
| | Yes | 0.461 (0.361–0.589) | | |
| | No | Reference[b] | | |
| Lack of research skills | | | | |
| | *Working hours* | | <0.001 | 0.024 |
| | < 20 | 1.481 (1.177–1.864) | | |
| | 20–30 | 1.431 (1.146–1.787) | | |
| | 31–40 | 1.665 (1.358–2.042) | | |
| | > 40 | Reference[b] | | |

[a] Nagelkerke $R^2$; [b] In logistic regression, one level of the independent variable serve as reference against which the odds of the other levels occurring are determined. 95% CI=95% confidence interval. Controlling for country.

## References

1. Jette DU, Bacon K, Batty C, Carlson M, Ferland A, Hemingway RD, et al. Evidence-based practice: Beliefs, attitudes, knowledge, and behaviors of physical therapists. Phys Ther. 2003;83(9).
2. Ferreira RM, Martins PN, Pimenta N, Gonçalves RS. Measuring evidence-based practice in physical therapy: a mix-methods study. PeerJ. 2022;9.
